# Synergistic treatment of linoleic acid and cefazolin on *Staphylococcus aureus* biofilm-related catheter infections

**DOI:** 10.1128/aem.00770-25

**Published:** 2025-05-21

**Authors:** Soyoung Ham, Han-Shin Kim, Min Jee Jo, Eunji Cha, Hwa-Soo Ryoo, Hyojin Kim, Heeho Lee, Gang-Jee Ko, Hee-Deung Park

**Affiliations:** 1 Department of Geosciences, University of Tübingen 9188, Tübingen, Germany; 2 Division of Biotechnology, College of Environmental and Bioresource Sciences, Jeonbuk National University 484934 , Iksan, Jeonbuk, Republic of Korea; 3 Department of Internal Medicine, Korea University Guro Hospital 211240 , Seoul, Republic of Korea; 4 Department of Civil, Environmental and Architectural Engineering, Korea University, Seoul, Republic of Korea; 5 KU-KIST Graduate School of Converging Science and Technology, Korea University, Seoul, Republic of Korea; Michigan State University, East Lansing, Michigan, USA

**Keywords:** synergistic combination, linoleic acid, cefazolin, *Staphylococcus aureus*, catheter biofilm

## Abstract

*Staphylococcus aureus* is a human pathogen that causes severe infections through biofilm formation. *S. aureus* biofilm is particularly susceptible to catheters in patients undergoing peritoneal dialysis. Although antibiotics are used to treat catheter infections, high-concentration treatments adversely affect human host immune systems and change the physicochemical properties of the catheters. To improve therapeutic outcomes without side effects, we combined antibiotics and natural products. In this study, we propose a combination of linoleic acid (LA) and cefazolin (CFZ) to treat *S. aureus* infections synergistically and apply it to catheter environments and *in vivo* systems. LA is a polyunsaturated fatty acid derived from natural products, and CFZ is a major antibiotic used to treat *S. aureus* catheter-related infections. The optimum synergistic condition was determined using silicon pad-forming biofilm similar to catheter materials. *S. aureus* biofilms were considerably inhibited *in vitro* and *in vivo* owing to the improved antibacterial effects. Furthermore, the combination negatively regulated the chemokine levels in the peritoneum, kidney, and liver extracted from mouse models. Moreover, it did not affect the cytotoxicity of human omentum mesothelial cells and the functions of the kidney and liver. Therefore, the combination of LA and CFZ could be a potential synergistic therapy for *S. aureus* catheter infections.

**IMPORTANCE:**

Catheter contamination is commonly caused by *Staphylococcus aureus* biofilm formation, primarily in peritoneal dialysis patients. Although antibiotics are used to treat catheter infections, high concentrations of antibiotics impair the immune system of the human host and alter the physicochemical properties of catheters. Therefore, it is crucial to improve therapeutic outcomes while minimizing the side effects of antibiotics. Combined treatments with natural products can be solutions to alleviate these problems. Our study offers a new synergistic combination (linoleic acid and cefazolin) for the control of catheter infections caused by *S. aureus* biofilms, especially in peritoneal dialysis.

## INTRODUCTION

*Staphylococcus aureus* is a major human pathogen frequently detected in the human body, colonizing approximately 30% of the human population (1). *S. aureus* is the most common cause of infections in hospitalized patients with various clinical diseases (e.g., peritonitis, endocarditis, osteoarticular, skin and soft tissue, and pleuropulmonary infections) (2). In particular, *S. aureus* is involved in medical device-associated infections. *S. aureus* can attach to the surfaces of implanted medical devices (e.g., catheters, artificial heart valves, and joint prosthetics), leading to considerable increases in morbidity and mortality through biofilm formation (3). *S. aureus* has an extraordinary capacity to attach to the polymer surfaces of medical devices or interact with human matrix proteins after the proteins cover the devices (4). Its capacity produces *S. aureus* biofilms, which are sticky aggregates embedded in an extracellular polymeric substance (EPS) consisting of proteins, carbohydrates, and extracellular DNA (3, 4). The *S. aureus* biofilm formation on medical devices increases resistance to antimicrobial drugs, contributing to the chronicity of infection (5).

Catheters, which are thin tubes that can be inserted into humans, are especially vulnerable to microbial contamination compared to other medical devices (6). As their surfaces are easily covered with host proteins, they provide sites for microbial attachment. Catheters are generally infected by microbes during the insertion or infusion of contaminated catheters and fluids (6, 7). Coagulase-negative staphylococci are the most frequent microbes in catheter-related infections, followed by *S. aureus*, enterococci, and *Candida* species (8). While *S. aureus* accounts for approximately 10% of etiologies associated with catheter-related infections, these organisms are regarded as significant pathogens due to their high degree of pathogenicity and the emergence of drug-resistant *S. aureus*, including methicillin-resistant *S. aureus* (MRSA) and vancomycin-resistant *S. aureus* (9). Once the catheter is contaminated, its removal is considered the simplest solution; however, catheter removal and reinsertion can cause life-threatening complications (10). Avoiding contamination of the catheter with bacteria or completely eradicating the bacteria after contamination is essential to prevent the recurrence of infection.

Catheter-related infections have their origins in various body sites, including the urinary tract, central venous, and peritoneum (11). Peritoneal dialysis (PD), a treatment modality that utilizes the peritoneal membrane to remove toxins and excess fluids, is a therapeutic approach employed in the management of end-stage kidney failure. The utilization of a peritoneal catheter for fluid exchange is imperative for the continuation of PD treatment (12). The greatest risk factors associated with peritoneal catheter maintenance include peritonitis and the formation of biofilms. Failure to properly manage and maintain the peritoneal catheter after peritonitis can result in frequent hospitalizations and technical complications (13). *S. aureus* has been identified as the second most common cause of PD-associated peritonitis (6). In particular, MRSA peritonitis is an independent predictor of permanent hemodialysis transfer and associated with a high risk of hospitalization (14). The poor outcome of *S. aureus* peritonitis is due to its high virulence, which causes severe complications (e.g., encapsulation of peritoneal adhesions and high chances of biofilm formation). Consequently, minimizing biofilm formation after *S. aureus* peritonitis can be a pivotal strategy to enhance outcomes.

Antibiotics are generally prescribed when a catheter is exposed to bacterial infections. However, the prescription of persistent and high concentrations of antibiotics can alter the desired physicochemical properties of the catheter (15). Moreover, antibiotic treatments often have adverse effects on the host’s immune system (15). Various technologies have been developed to address these problems and achieve maximal therapeutic effects for catheter biofilm control. Many researchers have searched for appropriate combinations as alternatives to single antibiotic treatments (16). Natural products are considered breakthroughs in the development of safe antibiofilm drugs for human health (17). However, they cannot effectively eliminate biofilms in clinical applications; therefore, natural products are often combined with conventional antibiotics (18). The combination of natural products and antibiotics addresses most drawbacks of antibiotics, including poor tissue penetration and slow bacterial death (18). We have previously introduced linoleic acid (LA) as an *S. aureus* biofilm inhibitor. LA, a polyunsaturated fatty acid, is non-toxic and widely distributed in plants, fruits, and oils (19). LA affects the biofilms of various gram-positive and -negative bacteria, including *S. aureus*. We found that LA inhibits biofilm formation by activating diffusible signal factor-mediated quorum sensing (20).

This study investigated the effects of LA and cefazolin (CFZ), one of the most frequently prescribed antibiotics, on the *S. aureus* catheter biofilm formation, and the optimal combined conditions were determined by extensive treatments with LA and CFZ. Silicon pads, with the same material as the catheter, were used for *S. aureus* biofilm studies to adjust the optimal combination to the catheter environment. The biofilm formation on the pads was analyzed using crystal violet staining and a drip-flow reactor. Furthermore, the pads were inserted into mouse models, and the chemokine and cytokine levels in the extracted organs were evaluated. In addition, the cytotoxicity of LA in the human omentum mesothelial cells was assessed, and the liver and renal functions were monitored following LA treatment.

## RESULTS

### Effects of LA and CFZ on *S. aureus*

**Fig 1:**
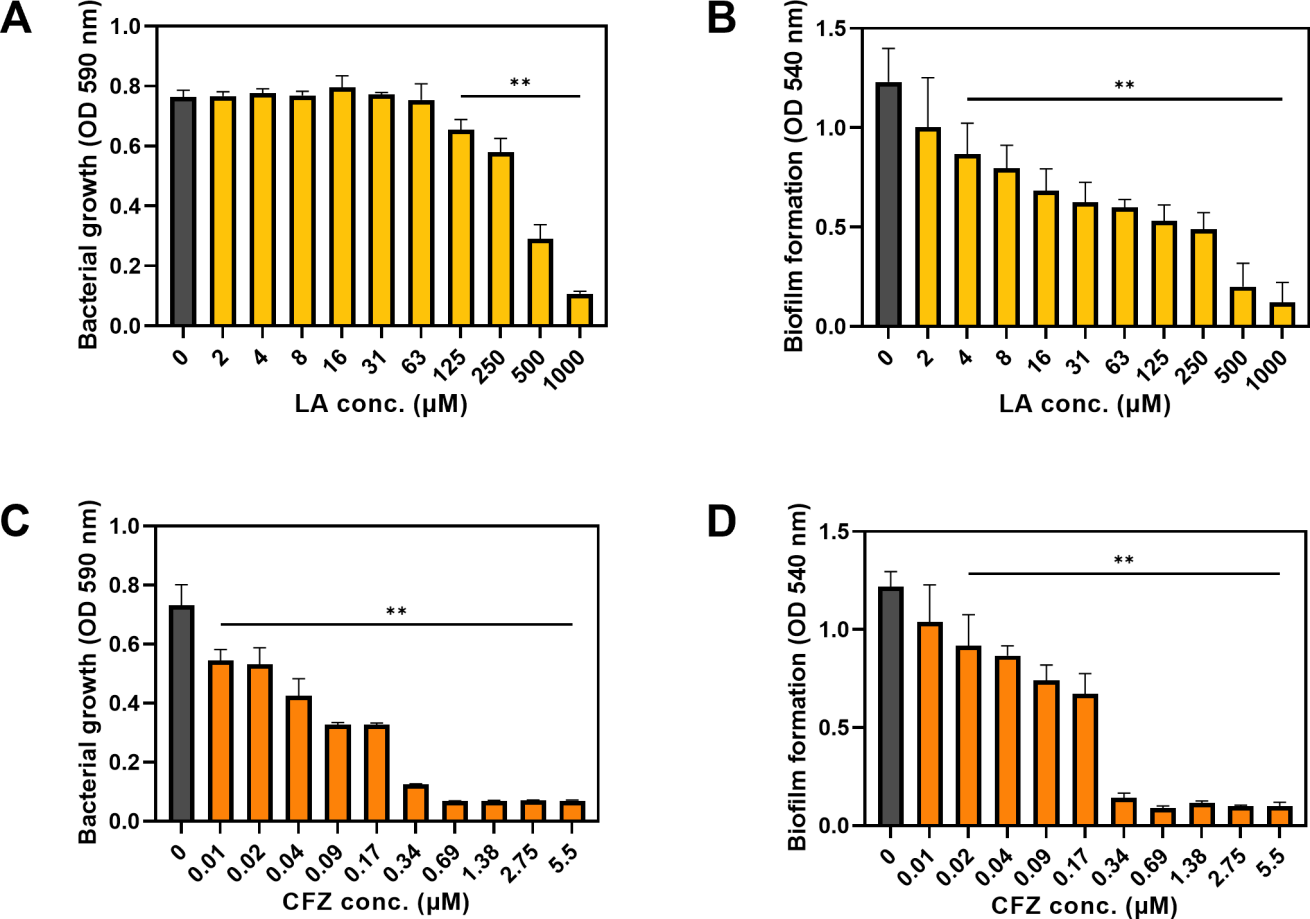
Bacterial growth and biofilm formation against *S. aureus* at various linoleic acid (LA) and cefazolin (CFZ) concentrations in a 96-well plate. (**A**) Bacterial growth and (**B**) biofilm formation by LA treatment (0–1,000 µM). (**C**) Bacterial growth and (**D**) biofilm formation by CFZ treatment (0–5.5 µM). (**) *P* < 0.005 compared with the control.

The effects of LA and CFZ on *S. aureus* were investigated by measuring the optical density (OD) at 590 and 540 nm for the bacterial growth and biofilm formation, respectively. The growth of *S. aureus* was not affected at low LA concentrations; however, it began to decrease rapidly with LA treatment at >125 µM, which is regarded as a minimal growth-affecting concentration (Fig. 1A). Consequently, the formation of the biofilm was suppressed by 57–90% at concentrations above 125 µM, attributable to antibacterial effects (Fig. 1B). Of particular interest is the observation that the biofilm formation was reduced by 19–51%, even at concentrations below 125 µM. Furthermore, when the OD for the biofilm formation was divided into that of bacterial growth, the normalized biofilm formation (OD 540/590 nm) decreased in response to the LA concentrations (Fig. S1A). This finding suggests that low concentrations of LA impact the *S. aureus* biofilm formation, potentially through mechanisms related to the *S. aureus* biofilm formation, without affecting the bacterial growth.

In contrast to LA, the overall concentration of CFZ affected the *S. aureus* growth, and the patterns were identical to those of the biofilm formation (Fig. 1C and D). When treated with >0.34 µM of CFZ on *S. aureus*, only 9–17% of bacterial growth and 7–12% of biofilm formation were observed compared to the control (i.e., without CFZ treatment). Furthermore, the normalized biofilm formation exhibited no significant disparities compared to the control due to comparable inhibition ratios between biofilm formation and bacterial growth (Fig. 1C and D; Fig. S1B). This result indicates that the *S. aureus* biofilm formation is highly susceptible to CFZ owing to its antimicrobial activity, which is similar to the results of other studies (21, 22).

### Synergistic effects of the combination of LA and CFZ on *S. aureus* biofilm

**Fig 2:**
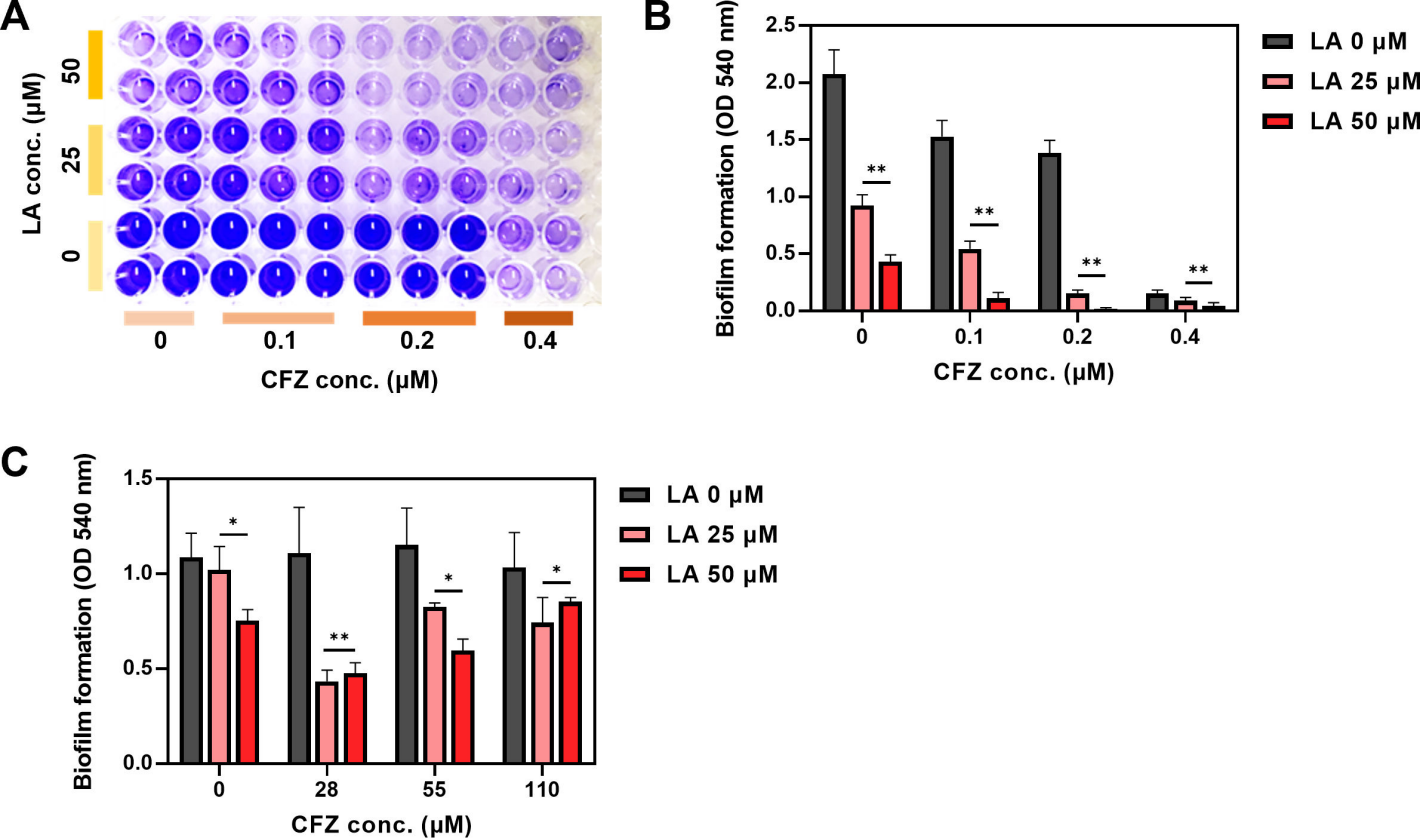
*S. aureus* biofilm formation and eradication by LA and CFZ combinations in a 96-well plate. (**A**) Image of crystal violet-stained biofilm cells. (**B**) Biofilm formation at different combinations of LA (0–50 µM) and CFZ (0–0.4 µM). (**C**) Biofilm eradication at different combinations of LA (0–50 µM) and CFZ (0–110 µM). (*) *P* < 0.05 and (**) *P* < 0.005 compared with the control.

The synergistic effects of LA and CFZ were evaluated by serial dilutions and biofilm staining with crystal violet in a 96-well plate (Fig. 2A). Lower concentrations of LA (0, 25, and 50 µM) and CFZ (0, 0.1, 0.2, and 0.4 µM) were used for the synergy test (Fig. 2B). Single treatment with LA or CFZ resulted in similar biofilm inhibition ratios, as shown in Fig. 1B and D. The biofilm formation was reduced by 55–79% when treated with LA alone; however, combined treatment with CFZ enhanced *S. aureus* biofilm inhibition ratios to 64–93% for 0.1 µM CFZ and 89–99% for 0.2 µM CFZ. This result indicates that the combined treatment with LA and CFZ had synergistic effects on the *S. aureus* biofilm inhibition. All LA combinations with 0.1 and 0.2 µM CFZ demonstrated synergistic responses according to the FICI (Table 1). Conversely, 0.4 µM CFZ exhibited additive effects in conjunction with LA. This synergism appears to involve improved antibacterial activity against *S. aureus*. Single treatments of 0–50 µM LA did not affect bacterial growth (Fig. S2A). However, when LA was combined with 0.1–0.4 µM CFZ, there was a 6–77% decrease in growth and similar inhibition ratios of the biofilm formation, contributing to an unvaried normalized biofilm formation (Fig. 2B; Fig. S2B). Concurrently, the combination treatment exhibited a marked increase in the killing rate of *S. aureus* in comparison to the individual treatments across a substantial range of LA and CFZ concentrations (Fig. S3A and B). The observed synergism appears to be specific to *S. aureus*, because overall concentrations of LA and CFZ did not reduce the biofilm formation by coagulase-negative staphylococci and enterococci (e.g., *Staphylococcus warneri* and *Enterococcus durans*), the most prevalent microbes in catheter-related infections (Fig. S4A and B, S5A and B) (8). As anticipated, no synergies for *S. warneri* and *E. durans* were observed with the same combined concentrations of LA and CFZ utilized in *S. aureus* (Fig. S4C and S5C).

**TABLE 1:** Synergistic interactions between LA and CFZ by fractional inhibitory concentration index (FICI)

Biofilm formation environment	Combination no.	Concentration (μM)		FICI	
		LA	CFZ	Value	Effect
96-well plate^*a*^	1	25	0.1	0.17	Synergy
	2	25	0.2	0.31	Synergy
	3	25	0.4	0.60	Additivity
	4	50	0.1	0.19	Synergy
	5	50	0.2	0.34	Synergy
	6	50	0.4	0.63	Additivity
Silicon pad^*b*^	1	25	0.2	0.10	Synergy
	2	25	0.3	0.13	Synergy
	3	25	0.4	0.17	Synergy
	4	50	0.2	0.12	Synergy
	5	50	0.3	0.16	Synergy
	6	50	0.4	0.20	Synergy

^*a*^ LA (1,000 µM) and CFZ (0.69 µM) were used as the minimum biofilm inhibitory concentration (MBIC) (Fig. 1B and D).

^*b*^ LA (1,000 µM) and CFZ (2.75 µM) were used as the MBIC (Fig. S7A and B).

Furthermore, the combination of LA and CFZ exhibited positive effects on the eradication of *S. aureus* biofilms (Fig. 2C). Higher concentrations of CFZ (0–110 µM) were administered to *S. aureus* biofilms compared to those for the biofilm formation (Fig. 2B). It is noteworthy that the eradication of established biofilms poses a greater challenge than the inhibition of the biofilm formation, given the fact that biofilms can increase antibiotic resistance by a factor of 10 to 1,000 (23). The eradication of biofilms was not achieved using single CFZ treatments, whereas LA eradicated *S. aureus* biofilms at 50 µM (Fig. 2C). The combination of LA and CFZ exhibited a synergistic effect; however, the biofilm eradication pattern was not as clear as that observed in the biofilm formation test (Fig. 2B).

### Application of the combination of LA and CFZ to catheter environments

**Fig 3:**
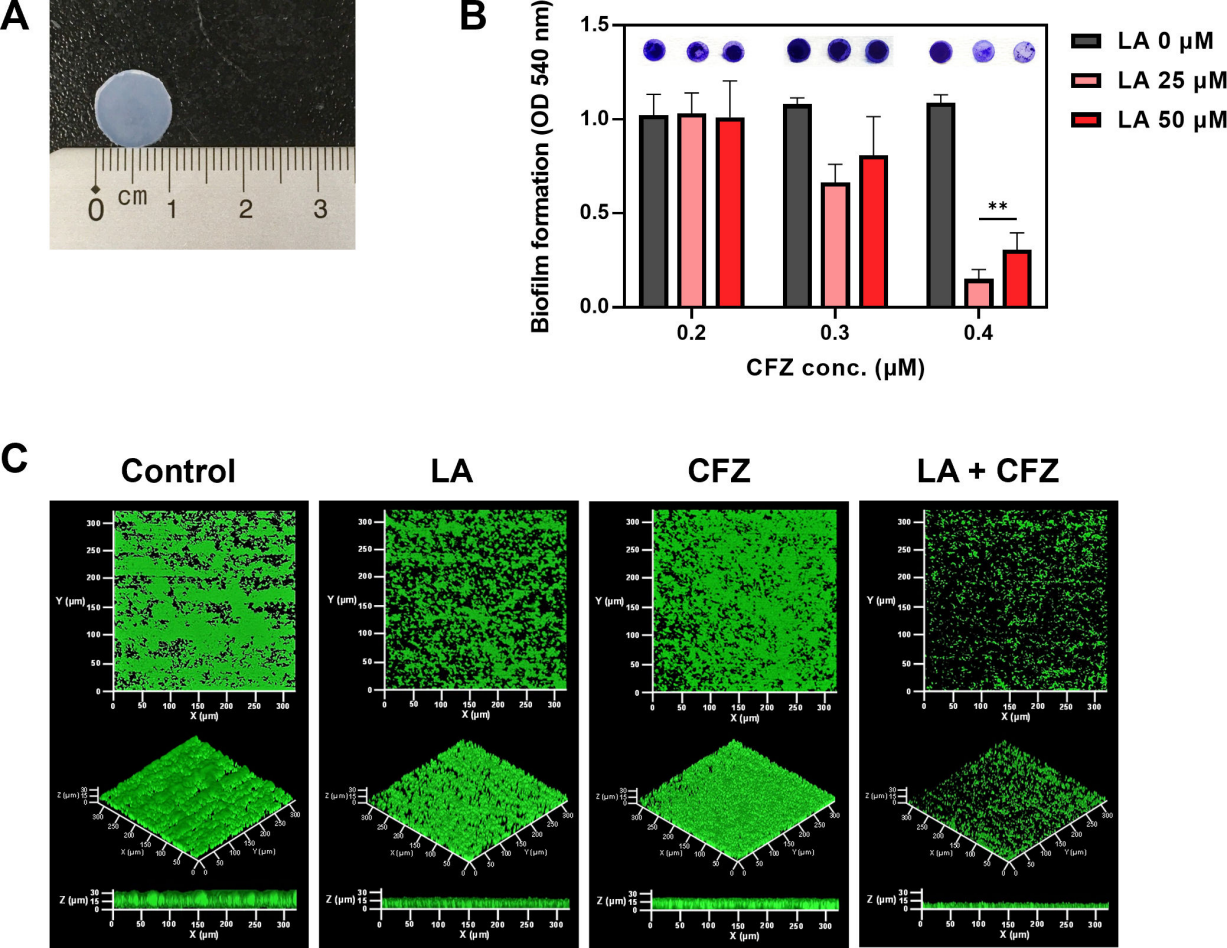
*S. aureus* biofilm formation by LA and CFZ combinations on silicon pads. (**A**) Silicon pads used in this study. (**B**) *S. aureus* biofilm formation at different combinations of LA (0–50 µM) and CFZ (0.2–0.4 µM) under static conditions. (**C**) Biofilm formation by the optimal combination of LA (25 µM) and CFZ (0.4 µM) under continuous conditions. (**) *P* < 0.005 compared with the control.

A wide range of polymers, including silicone, polyurethane, polyethylene, and terephthalate, are available as catheters (24). Silicone is one of the most implantable materials due to its flexibility, safety, and biocompatibility (25). Accordingly, the effects of the combination of LA and CFZ on catheter biofilms were investigated using silicone pads, similar to catheter materials (Fig. 3A). The biofilm formation on the silicon pads was measured under static and flow conditions.

Interestingly, when the same concentration of LA (0–50 µM) or CFZ (0–0.4 µM) treatment in Fig. 2B was applied to the *S. aureus* biofilms formed on the silicon pad, no apparent biofilm inhibitory effects were observed (Fig. 3B). Overall, higher CFZ concentrations were required for synergism under the silicon pad conditions (Fig. 2B and 3B). Combined treatment with 25 µM LA and 0.4 µM CFZ resulted in the highest biofilm inhibitory activity (86%) among all combinations. Furthermore, the colony numbers of the biofilm cells treated with the combination were the lowest compared to those of single treatments (Fig. S6). The combination exhibited true synergism, with a value of 0.17 for the FICI, indicating that the synergism did not originate from additive effects (Fig. S7A and B; Table 1). Therefore, the concentration (25 µM LA and 0.4 µM CFZ) was determined to be an optimal synergistic condition for the subsequent investigation of catheter biofilm studies.

The *S. aureus* biofilm formation at the optimal combination of LA and CFZ (25 and 0.4 µM, respectively) was observed using a drip-flow reactor, as this system can model the environment of catheters as a way to grow a biofilm under low shear and air-liquid interface conditions (26). The control biofilm (i.e., without treatment) retained typical spherical shapes with 30 µm^3^/µm^2^ of volume and 34 µm of thickness (Fig. 3C; Fig. S8A). The CFZ-treated biofilm was similar to the control biofilm. In contrast, LA suppressed the volume and thickness of the biofilm by up to approximately 41% with loose and sparser shapes. However, no significant differences were observed under the static conditions (Fig. 3B). Combined treatment with LA and CFZ changed the biofilm remarkably, with 70% volume and 58% thickness reduction of the biofilm compared to the control (Fig. S8A). Moreover, carbohydrates and proteins, which are the main components of EPS in biofilms, showed patterns similar to those in the CLSM images (Fig. 3C; Fig. S8B) (3, 4). Biofilms treated with the combination showed a high inhibitory activity against EPS components (27–59%). This result suggests that the combination of LA and CFZ may synergistically alleviate the *S. aureus* catheter biofilms under both static and flow conditions.

### Application of the combination of LA and CFZ in an *in vivo* system

**Fig 4:**
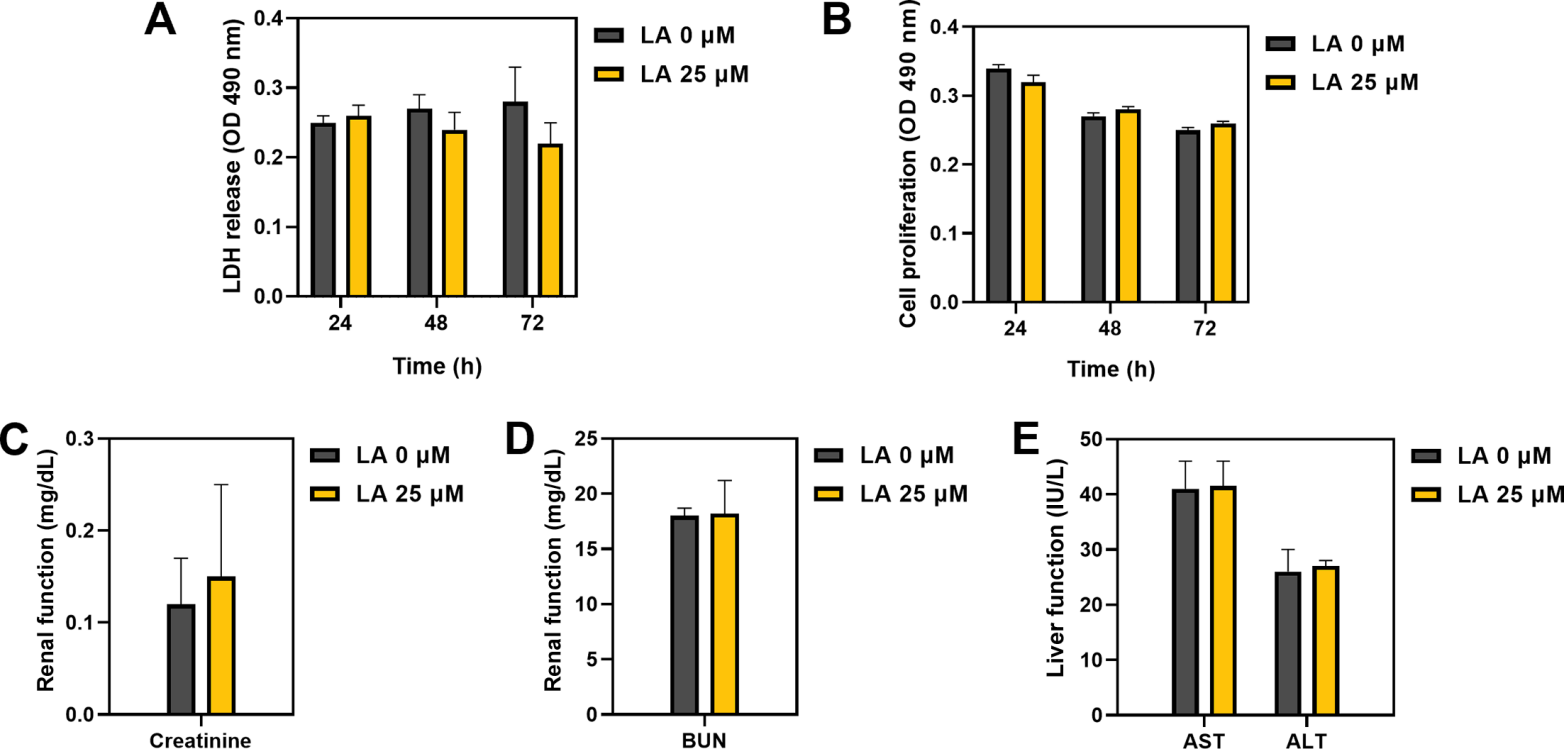
Bioavailability of LA (25 µM) on human mesothelial cells obtained by the primary cell culture from the omentum. (**A**) Lactate dehydrogenase (LDH) and (**B**) MTS cell proliferation using colorimetric methods. (**C**) Creatinine and (**D**) blood urea nitrogen (BUN) levels to monitor the renal function. (**E**) Aspartate transaminase (AST) and alanine transaminase (ALT) levels to monitor the liver function.

To analyze its bioavailability, the cytotoxicity of LA was examined using *in vitro* and *in vivo* experiments. In the context of *in vitro* experimentation, the levels of lactate dehydrogenase (LDH) and cell proliferation were evaluated following 24, 48, and 72 h of exposure to 25 µM LA treatment by utilizing omentum-derived mesothelial primary cells. Compared with the control (i.e., untreated cells), LA showed no remarkable differences in LDH release and cell proliferation below 20% under all conditions (Fig. 4A and B).

For the *in vivo* experiments, the kidney and liver functions were measured in mice after the intraperitoneal administration of LA. Creatinine and blood urea nitrogen (BUN) were measured to analyze nephrotoxicity (Fig. 4C and D). Although creatinine levels increased by 25% after LA treatment, the changes were not significant. The creatinine levels in both groups remained within the normal range for the kidney function. Furthermore, no changes were observed in BUN between the control and LA-treated groups. The aspartate transaminase (AST) and alanine transaminase (ALT) levels were measured to assess hepatotoxicity (Fig. 4E). The AST and ALT levels did not differ between the control and LA-treated groups. This suggests that LA can be safely used in clinical settings.

**Fig 5:**
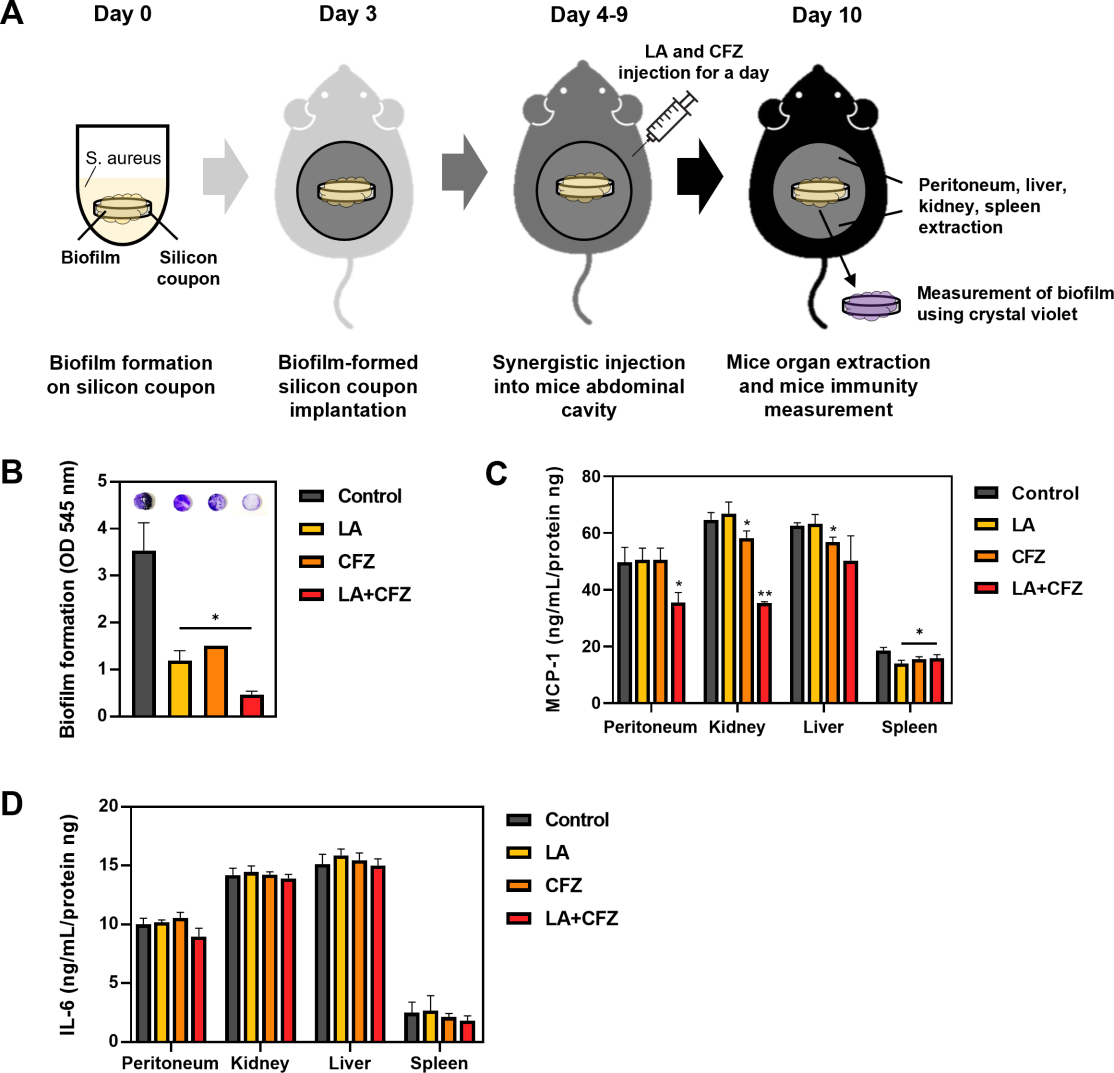
Mouse models for testing *S. aureus* infections by the optimal combination of LA (25 µM) and CFZ (0.4 µM). (**A**) Schematic diagram of the methodology. (**B**) Biofilm formation on silicon pads obtained from mice. (**C**) Chemokine and (**D**) cytokine levels extracted from mice organs. (*) *P* < 0.05 and (**) *P* < 0.005 compared with the control.

To analyze the synergistic effects of LA and CFZ on the *S. aureus* biofilm formation and inflammation, silicon pads covered with *S. aureus* biofilms were implanted into the peritoneal cavity and treated with an intraperitoneal administration of vehicle, LA, CFZ, or their combination for 1 week (Fig. 5A). Single LA or CFZ treatments successfully suppressed the biofilm formation from the extracted pads by 57–66%. Combined treatment with LA and CFZ inhibited most of the biofilms by 87% (Fig. 5B), consistent with the results in Fig. 3.

The degree of peritoneal inflammation was measured in mouse organs after the administration of vehicle, LA, CFZ, or their combination with the *S. aureus* biofilm-coated silicon pad implantation. The degree of inflammation after silicon implantation and administration of LA and CFZ was measured based on the levels of proinflammatory cytokines and chemokines (i.e., monocyte chemoattractant protein-1 [MCP-1] and interleukin-6 [IL-6]), which were induced in the peritoneum by *S. aureus* (27, 28). The combination of LA and CFZ inhibited the production of MCP-1 in the peritoneum, kidney, liver, and spleen by 15–29% (Fig. 5C). Accordingly, 1–27% of IL-6 production was negatively affected by the combination, although the changes were not statistically significant (Fig. 5D). Therefore, combination treatment with LA and CFZ seems to have synergistic effects on the mitigation of the biofilm formation and the peritoneal inflammation caused by *S. aureus*.

## DISCUSSION

This study unequivocally demonstrated the synergistic effects of natural products and antibiotics on *S. aureus* catheter biofilms. In a similar vein, Bonincontro et al. observed the synergistic effects of natural compounds combined with antimicrobials against *S. aureus* biofilms (29). The observed synergism resulted in the attainment of adequate therapeutic effects at low doses. Zacchino et al. introduced natural products that potentiate the antibiofilm capacity of antimicrobial drugs (30). In addition to these studies, numerous researchers have directed their efforts toward identifying novel synergistic combinations and elucidating their mechanisms of action (31, 32).

We used LA as a natural product and CFZ as an antibiotic to synergistically treat *S. aureus* catheter biofilms. Penicillin is an anti-staphylococcal antibiotic that is preferred by patients because of its narrow spectrum and high efficiency compared to other antimicrobial agents. However, owing to concerns regarding frequent dosing, expensive acquisition costs, and side effects, CFZ is increasingly used as an alternative (33, 34). CFZ, a first-generation cephalosporin, enables the treatment of *S. aureus* infections with fewer clinical failures (34). Depending on its antibacterial activity, CFZ is expected to effectively control infections by reducing biofilms (Fig. 1C and D). LA demonstrated antibacterial activity at high concentrations, while in low concentrations, LA exhibited the capacity to impede the *S. aureus* biofilm formation without compromising bacterial growth (Fig. 1A and B). The detailed molecular mechanisms of LA are yet to be elucidated but may be related to fatty acids (35). The amphipathic properties of fatty acids destroy cell membranes at high concentrations, whereas interference with bacterial signaling is assumed to be involved in the *S. aureus* biofilm formation at low concentrations.

The synergistic biofilm control capacity of the combination originated from its enhanced antimicrobial activity (Fig. S2A). However, we still need to find detailed synergistic mechanisms. Chan et al. reported that LA can be used as an antibiotic adjuvant (36). They speculated that this synergism was probably related to the efflux MsrA pump interference in *S. aureus*. Hess et al. suggested that this effect is due to the facilitation of the biofilm matrix penetration by the surfactant properties of LA (37). We also observed enhanced biofilm control efficiency of antimicrobial agents when combined with LA (38). Biofilm loosening by LA treatment may allow antimicrobial agents to penetrate biofilms more easily and rapidly (38). Although some studies have described the molecular mechanisms responsible for this synergism, it is currently difficult to draw clear conclusions (35). However, LA capabilities, such as alteration of the permeability, morphology, and stability of cell membranes, may eventually contribute to improved antimicrobial efficiency by inducing antibiotic uptake (39).

In order to facilitate the clinical application of the LA and CFZ combination, it is necessary to elucidate the effects of this combination on both the pathogenic and immune systems. Catheters are susceptible to microbial colonization among other devices, accounting for more than 60% of hospital-acquired infections (40). Given its mechanical properties and biocompatibility, silicon is the primary material used in catheters, especially for patients undergoing PD (41). However, the hydrophilization of silicon surfaces promotes bacterial adhesion and biofilm formation through constant contact with the human body and bodily fluids (42). Furthermore, the sensitivity of *S. aureus* to antibiofilm agents is known to decrease by 2- to 10-fold when grown in contact with silicon catheters (43).

Bhattacharya et al. indicated that LA positively influences inflammatory response and immune functions (44). However, it is imperative to note that high concentrations of LA (>100 µM) have been observed to exert deleterious effects on diverse cell lines (45–47). Moran et al. found that LA is toxic in a rabbit renal proximal tubule model by inducing mitochondrial dysfunction before cell death (48). The potential for LA to induce a cytotoxic response can be mitigated by combining it with CFZ, as synergistic effects were observed at much lower concentrations of LA required for a single-agent activity (Fig. 2B and 3B). Nevertheless, the continuous administration of LA has the potential to be detrimental to patients. Consequently, the close monitoring of any potential side effects is crucial to ensure the safety of patients.

Several researchers have introduced various combinations to *in vivo* systems for treating catheter-related infections. Liu et al. inserted MRSA biofilm-formed silicon into a rat model and measured infectious levels (49). The combination of linezolid and baicalein led to a significant reduction in CFU counts, *Staphylococcus aureus* enterotoxin A, C-reactive protein, and procalcitonin when compared with single treatments. Linezolid has been shown to enhance the therapeutic efficacy of other antibiotics, such as fosfomycin, levofloxacin, and rifampin, in a rat model of MRSA infection (50). Furthermore, El-Rehewy et al. coated catheters with ciprofloxacin and N-acetyl cysteine through an instant dip method. The catheters that were coated with this combination exhibited not only prolonged antimicrobial durability but also high removal efficiency against *S. aureus* (51). However, for practical use in clinical settings, combinations of these agents must undergo rigorous monitoring and evaluation through clinical trials on patients. If the clinical application of these combinations is addressed in the future, they can be provided as new therapies for *S. aureus* biofilm-related catheter infections.

Combined treatment with LA and CFZ significantly attenuated the *S. aureus* catheter biofilm formation compared to single treatments both *in vitro* and *in vivo*. The combination inhibited and eradicated the *S. aureus* biofilms formed on the silicon pads, similar to catheter materials, by increasing antibacterial activity. The combination also showed synergistic effects on chemokine and cytokine levels in organs extracted from mouse models. Furthermore, the cytotoxicity of the human omentum mesothelial cells and the functions of the kidney and liver remained stable, even after LA treatment. Therefore, the synergistic combination of LA and CFZ could potentially be used as a safe and effective therapeutic approach for controlling *S. aureus* catheter biofilms.

## MATERIALS AND METHODS

### Bacterial strains and chemicals

This study was performed using the model microorganism, *S. aureus* ATCC6538. The microorganisms were grown in a shaking incubator at 37°C and 250 rpm using tryptic soy broth (TSB) (BD Difco, Detroit, MI, USA). LA [(9Z, 12Z)-octadeca-9, 12-dienoic acid] and CFZ were purchased from Sigma-Aldrich (St. Louis, MO, USA). LA was dissolved in 99.9% dimethyl sulfoxide (Carl Roth, Karlsruhe, Germany), and deionized water was used for CFZ.

### Static biofilm formation test

Static biofilm formation tests were performed using broth microdilution. First, 100 µL of TSB was added to each well of a 96-well plate (Sigma-Aldrich). LA and CFZ were added to the wells in concentration ranges of 0–1,000 and 0–5.5 µM, respectively, through twofold serial dilutions. Next, 100 µL of *S. aureus* (OD at 590 nm = 0.01) was added to wells in the plate and incubated at 37°C for 24 h without agitation. Bacterial growth was determined by measuring the OD at 590 nm using an iMark microplate reader (Bio-Rad, Richmond, CA, USA). Subsequently, the planktonic cells in the wells were discarded, and the remaining cells were washed twice with phosphate-buffered saline (PBS; 137 mM NaCl, 2.7 mM KCl, 10 mM Na_2_HPO_4_, and 2 mM KH_2_PO_4_, pH 7.2). The biofilm cells were stained with 0.1% crystal violet solution for 10 min. After washing using deionized water, the bounded crystal violet to the biofilm cells was eluted with 99.9% ethyl alcohol. The amount of the biofilm formation was quantified by measuring the OD at 540 nm using a microplate reader.

### Synergistic biofilm formation test between LA and CFZ

The synergistic effects were evaluated at various concentrations of LA and CFZ using two biofilm inhibition tests. First, a static biofilm formation test was performed. Next, 100 µL of TSB was added to each well of a 96-well plate. Subsequently, 100 µL of LA (0–50 µM) was distributed from top to bottom, while 100 µL of CFZ (0–0.4 µM) was distributed from the left to right of the plate. Then, 100 µL of *S. aureus* (OD at 590 nm = 0.05) was finally distributed to the wells, and the plate was incubated at 37°C for 24 h to form biofilm.

Second, a biofilm test was performed using silicon pads. Silicon identical to the material used in catheters was cut into circles (1 cm diameter) and inserted into a 12-well plate with *S. aureus* (OD at 590 nm = 0.05) and various concentrations of LA (0–50 µM) and CFZ (0.2–0.4 µM). The biofilms formed on the silicon pads were measured using methods similar to those employed for the static biofilm formation test. Briefly, the amount of biofilm formation was estimated after washing with PBS, staining with crystal violet, and elution with ethyl alcohol. The potential synergistic effects of LA and CFZ were analyzed by measuring the OD at 540 nm using a microplate reader.

### Fractional inhibitory concentration index

The interactions between LA and CFZ were substantiated through the use of fractional inhibitory concentration index (FICI) (52). The FICI was calculated as follows:


FICI = FIC (LA) + FIC (CFZ)

FIC (LA) is defined as the minimum biofilm inhibitory concentration (MBIC) of LA in combination/MBIC of LA alone. Similarly, FIC (CFZ) is defined as the MBIC of CFZ in combination/MBIC of LA alone. The MBIC was determined as the lowest concentration of LA or CFZ with <10% of the biofilm formation compared to the control (i.e., without treatment) (53). The interactions were classified based on the FICI as follows: synergy (FICI ≤ 0.5), additivity (0.5 < FICI ≤ 1), indifference (1 < FICI ≤ 2), and antagonism (FICI > 2).

### Synergistic biofilm eradication test between LA and CFZ

The synergistic effects for biofilm eradication were evaluated under various concentrations of LA (0–50 µM) and CFZ (0–110 µM). The mixture of 100 µL of TSB and 100 µL of *S. aureus* (OD at 590 nm = 0.01) was distributed to each well of a 96-well plate, and the plate was incubated at 37°C for 24 h to form biofilms. To induce biofilm eradication, 100 µL of LA (0–50 µM) was distributed from top to bottom, while 100 µL of CFZ (0–110 µM) was distributed from left to right of the plate, followed by incubation at 37°C for 24 h. The amounts of biofilms were measured using a similar method to the static biofilm formation test. The potential synergistic biofilm eradication effects between LA and CFZ were analyzed by measuring the OD at 540 and 590 nm using a microplate reader.

### Continuous biofilm formation test

Silicon pads attached to glass slides were inserted into a drip-flow biofilm reactor (BioSurface Technologies, Bozeman, MT, USA). TSB containing *S. aureus* (OD at 590 nm = 0.05) with single treatments of 25 µM LA or 0.4 µM CFZ and a combined treatment of 25 µM LA and 0.4 µM CFZ were continuously fed into the reactor using a peristaltic pump (Masterflex C/L tubing pumps, Cole-Parmer, Vernon Hills, IL, USA) at 20 mL/h. After operation at 37°C for 48 h, the attached biofilm cells were washed with PBS and stained with 4′,6-diamidino-2-phenylindole (Carl Roth) for 10 min. The stained biofilm cells were observed by confocal laser scanning microscopy (CLSM) (Carl Zeiss LSM700, Jena, Germany). Confocal images were captured with a 40× objective lens (C-Apochromat 40×/1.20 W Korr M27, Carl Zeiss) and observed in Z-stack mode using the Zen 2011 program (Carl Zeiss). The biofilms were quantified by measuring volume (μm^3^/μm^2^) and average thickness (μm) using CLSM images and Comstat2 in the ImageJ program (54).

### Generation of mesothelial primary cells

The mesothelial primary cells derived from the omentum of the tissue remaining after abdominal surgery were donated by Professor Kang (EUMC 2018-01-073) of Ewha University. The mesothelium was washed several times with PBS (Gibco, Grand Island, NY, USA) containing a 1% antibiotic-antimycotic solution (GenDEPOT, Barker, TX, USA). The specimen was finely minced and subjected to enzymatic disaggregation by incubation with 0.125% trypsin-EDTA (Gibco) for 30 min at 37°C. Following the incubation, the suspension was centrifuged at 50 ×*g* for 5 min and plated into collagen-coated tissue culture flasks with Ham’s F-12 medium containing 10% fetal bovine serum (Gibco).

### *In vitro* examination of LA cytotoxicity

To analyze the toxic effects of LA, cell viability was measured as the degree of cell proliferation using a CellTiter 96 Aqueous One Solution Cell Proliferation Assay Kit (G3580; Promega, Madison, WA, USA). Mesothelial primary cultured cells were seeded into a 96-well plate and treated with 25 µM of LA for 24, 48, and 72 h. After the incubation, cells were stained with 20 µL of 3-(4,5-dimethylthiazol-2-yl)-5-(3-carboxymethoxyphenyl)-2-(4-sulfophenyl)-2H-tetrazolium inner salt for 1 h. Absorbance was measured at 495 nm using a microplate reader (SpectraMax 190; Molecular Devices, CA, USA).

For an additional cytotoxicity analysis, the released LDH was measured using a CyQUANT LDH Cytotoxicity Assay Kit (Invitrogen, Carlsbad, CA, USA) according to the manufacturer’s instructions. Mesothelial primary cultured cells were seeded into a 96-well plate and treated with 25 µM of LA. The cells were lysed with lysis buffer for 45 min at 37°C. Following the incubation period, the supernatants were transferred, and the substrate mixture was added for the reaction. The reaction was terminated using a stop solution. The absorbance was measured using a microplate reader at 490 and 680 nm.

### *In vivo* examination of LA toxicity

Ten-week-old male C57BL/6 mice were treated with a vehicle and 25 µM of LA for 1 week. To analyze the effects of LA toxicity on organ functions, the kidney and the liver were examined. The serum BUN and creatinine levels were assessed to evaluate the kidney function. The AST and ALT levels were analyzed to identify the liver function using a biochemical analyzer (Fuji Dry-Chem NX 500i; Fujifilm, Kanagawa, Japan). All animal experiments were conducted according to animal care guidelines approved by the Institutional Animal Care and Use Committee of the Korea University College of Medicine (KOREA-2021-0010).

### Peritoneal cavity inflammation measurement

To investigate the actual synergistic effects of LA and CFZ *in vivo*, silicon pads covered with a 3 day-incubated *S. aureus* biofilm were implanted into the peritoneal cavity of mice. Each of the five mice given three silicon pads was treated daily with an intraperitoneal administration of vehicle, LA, CFZ, or their combination for 1 week. After sacrificing the mice, the silicon pads were collected to determine the degree of the biofilm formation. Peritoneal, liver, spleen, and kidney tissues were extracted. The degree of peritoneal inflammation and the levels of MCP-1 and IL-6 were measured in the peritoneum, liver, spleen, and kidney using a Mouse MCP1 enzyme-linked immunosorbent assay (ELISA) Kit (ab100722; Abcam, Cambridge, UK) and an IL-6 Mouse ELISA Kit (ab100713; Abcam) according to the manufacturer’s instructions. Briefly, the standard and samples were incubated for 2.5 h at 25°C. After a washing step, the samples were incubated with 1× biotinylated detection antibody for 1 h at 25°C with gentle shaking. Subsequently, 1× HRP-streptavidin solution was added to the samples for 45 min at 25°C. The samples were incubated with TMP one-step substrate reagent for 30 min at 25°C. Following the incubation period, a stop solution was added, and color changes were measured at 450 nm using a microplate reader.

## SUPPLEMENTAL MATERIAL

Supplemental figures aem.00770-25-s0001.docx Figures S1 to S8.

ASM does not own the copyrights to Supplemental Material that may be linked to, or accessed through, an article. The authors have granted ASM a non-exclusive, world-wide license to publish the Supplemental Material files. Please contact the corresponding author directly for reuse.
