# Supplementary material for: Synergistic treatment of linoleic acid and cefazolin on Staphylococcus aureus biofilm-related catheter infections
Source: Appl Environ Microbiol. 2025 May 21;91(6):e00770-25. doi: 10.1128/aem.00770-25 (PMC12175515; doi:10.1128/aem.00770-25)
Supplement: Supplemental figures — Figures S1 to S8. [file aem.00770-25-s0001.docx]

**Supplemental materials**

**Synergistic treatment of linoleic acid and cefazolin on *Staphylococcus aureus* biofilm-related catheter infections**

Soyoung Ham^a,1^, Han-Shin Kim^b,1^, Min Jee Jo^c^, Eunji Cha^d^, Hwa-Soo Ryoo^d^, Hyojin Kim^b^, Heeho Lee^b^, Gang-Jee Ko^c,^* and Hee-Deung Park^d,e,^*

^a^Department of Geosciences, University of Tübingen, Schnarrenbergstraße 94-96, Tübingen 72076, Germany

^b^Division of Biotechnology, College of Environmental and Bioresource Sciences, Jeonbuk National University, Iksan, Jeonbuk 54596, Republic of Korea

^c^Department of Internal Medicine, Korea University College of Medicine, Korea University Guro Hospital, Seoul 08308, Republic of Korea

^d^Department of Civil, Environmental and Architectural Engineering, Korea University, Seoul 02841, Republic of Korea

^e^KU-KIST Graduate School of Converging Science and Technology, Korea University, Seoul 02841, Republic of Korea

^1^Contributed Equally

^*^Corresponding Author

Hee-Deung Park, Ph.D.

Department of Civil, Environmental and Architectural Engineering, Korea University, Seoul 02841, Republic of Korea

Tel.: +82-2-3290-4861; Fax: +82-2-928-7656; E-mail: heedeung@korea.ac.kr

Gang-Jee Ko, Ph.D.

Department of Internal Medicine, Korea University College of Medicine, Korea University Guro Hospital, Seoul 08308, Republic of Korea

Tel.: +82-2-2626-3039; FAX: +82-2-2626-1076; E-mail: lovesba@korea.ac.kr

**Extracellular polymeric substances (EPS) production test**

The washed biofilm cells from the continuous biofilm formation test were lysed with 0.01M KCl using a sonicator (VCX 750, SONICS, Newtown, CT, USA) for 4 cycles of 5 s of operation and 5 s of pause at 20 % amplitude. The sonicated biofilm cells were filtered through a 0.22 μm membrane filter to measure carbohydrates and proteins in EPS. For carbohydrates, a mixture of 250 μL of filtrate and 750 μL of 99.9 % sulfuric acid (Junsei, Tokyo, Japan) was incubated at 25℃ for 30 min. Then, 150 μL of 5 % phenol was added to the mixture and incubated at 90℃ for 5 min. The amount of carbohydrates was quantified at OD at 490 nm using a microplate reader. For proteins, a mixture of 200 μL of filtrate and 1000 μL of Lowry reagent (Sigma-Aldrich) was incubated at 25℃ for 10 min. 100 μL of Folin-Ciocalteu reagent (Sigma-Aldrich) was added to the mixture and incubated at 25℃ for 30 min in the dark. The amounts of proteins were quantified by measuring OD at 750 nm using a microplate reader.


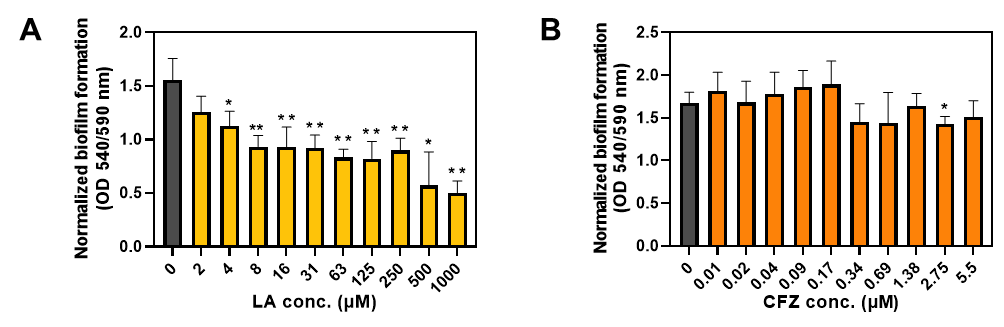


**Fig. S1.** Normalized biofilm formation against *S. aureus* at various linoleic acid (LA) and cefazolin (CFZ) concentrations in a 96-well plate. Normalized biofilm formation by (A) LA (0–1000 μM) and (B) CFZ (0–5.5 μM) treatments. The optical density (OD) of crystal violet-stained biofilm cells (OD 540 nm) was divided into that of bacterial growth (OD 590 nm). (*) P<0.05 and (**) P<0.005 compared with the control.


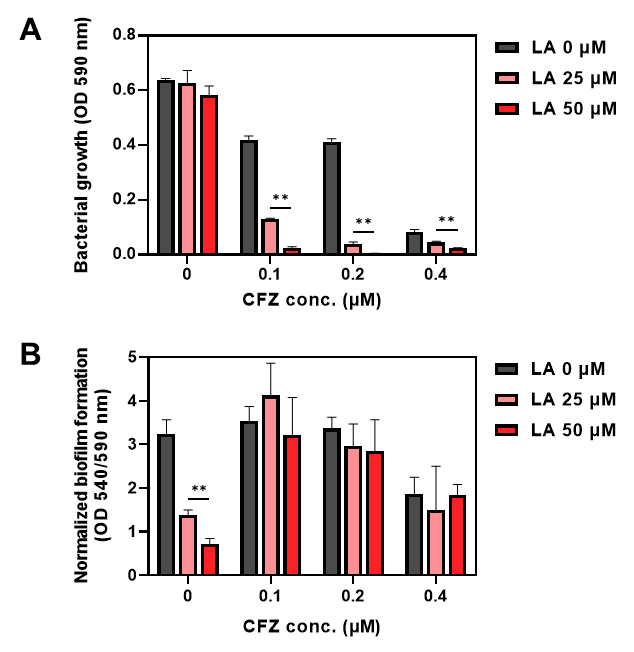


**Fig. S2.** Bacterial growth and normalized biofilm formation against *S. aureus* by LA and CFZ combinations in a 96-well plate. (A) Bacterial growth and (B) normalized biofilm formation at different combinations of LA (0–50 μM) and CFZ (0–0.4 μM). (**) P<0.005 compared with the control.


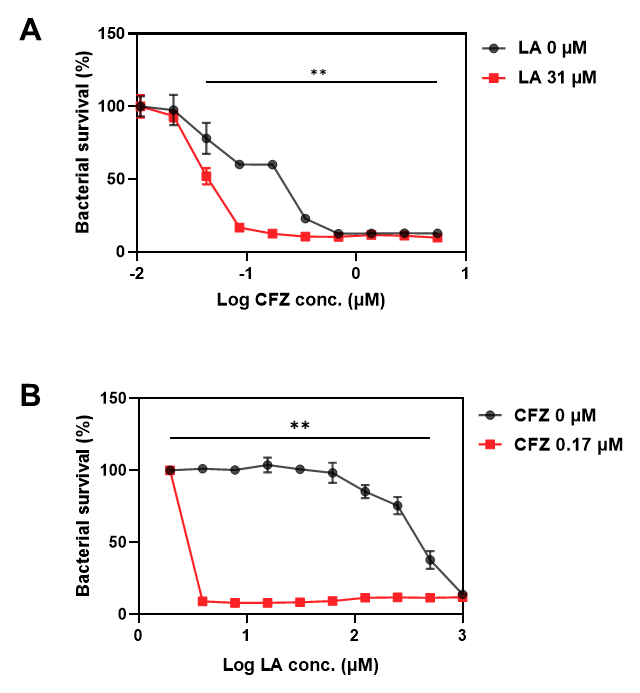


**Fig. S3.** Killing curves of *S. aureus* by single and combined treatments of LA and CFZ in a 96-well plate. (A) Bacterial survival by LA treatment (0, 31 μM) at various concentrations of CFZ (0–5.5 μM). (B) Bacterial survival by CFZ treatment (0, 0.17 μM) at various concentrations of LA (0–1000 μM). Bacterial survival was measured by OD at 590 nm. (**) P<0.005 compared with the control.


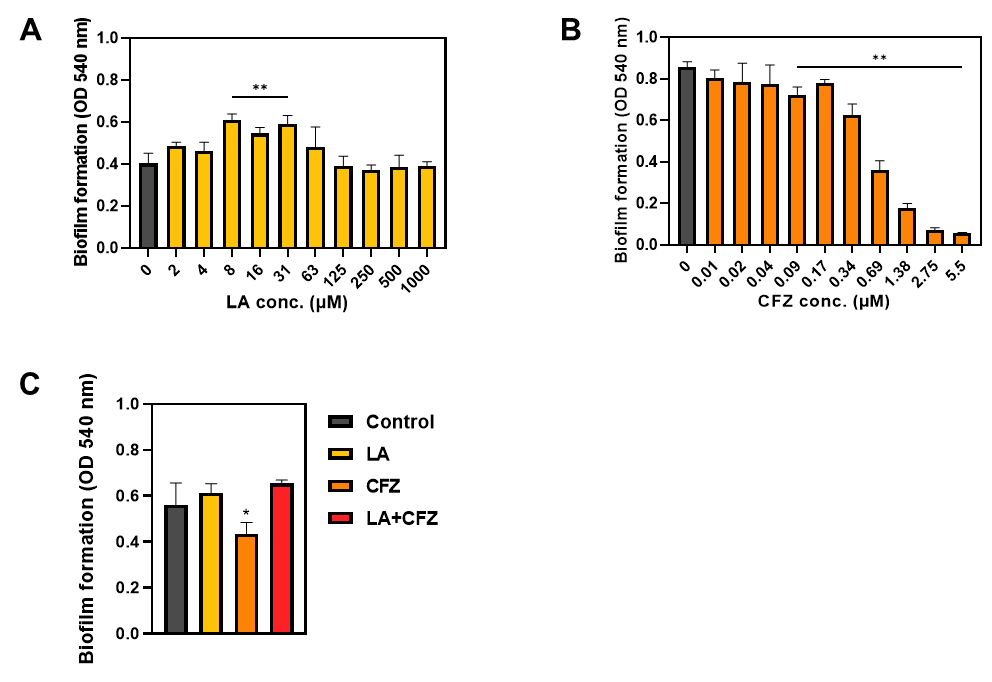


**Fig. S4.** Biofilm formation against *S. warneri* by single and combined treatments of LA and CFZ in a 96-well plate. Biofilm formation by (A) LA (0–1000 μM), (B) CFZ (0–5.5 μM), and (C) combination of LA (25 μM) and CFZ (0.4 μM). (*) P<0.05 and (**) P<0.005 compared with the control.


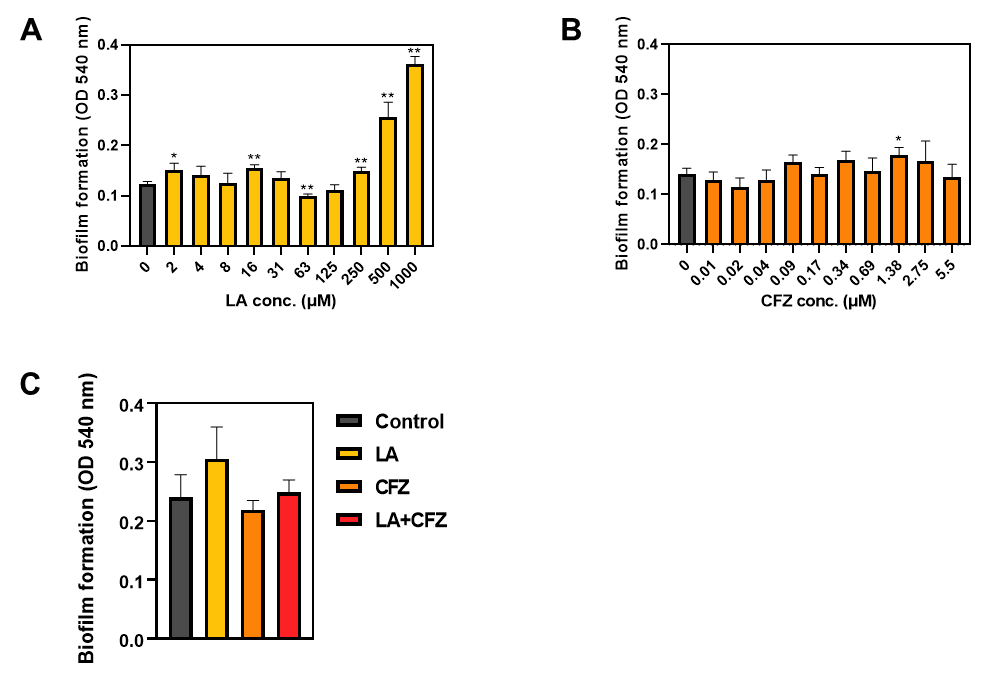


**Fig. S5.** Biofilm formation against *E. durans* by single and combined treatments of LA and CFZ in a 96-well plate. Biofilm formation by (A) LA (0–1000 μM), (B) CFZ (0–5.5 μM), and (C) combination of LA (25 μM) and CFZ (0.4 μM). (*) P<0.05 and (**) P<0.005 compared with the control.


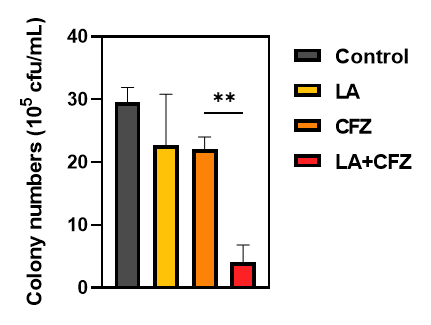


**Fig. S6.** Colony numbers of *S. aureus* biofilm cells by the optimal combination of LA (25 μM) and CFZ (0.4 μM). Biofilm was formed on silicon pads for 24 h. (**) P<0.005 compared with the control.


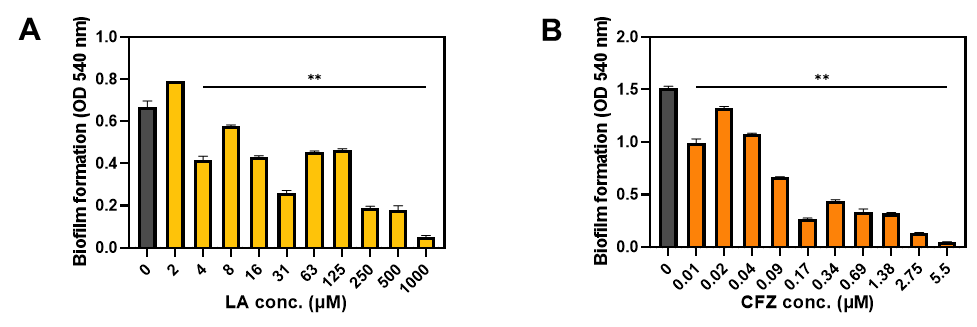


**Fig. S7.** Biofilm formation against *S. aureus* at various LA and CFZ concentrations on silicon pads. Biofilm formation by (A) LA (0–1000 μM) and (B) CFZ (0–5.5 μM) treatments. (**) P<0.005 compared with the control.


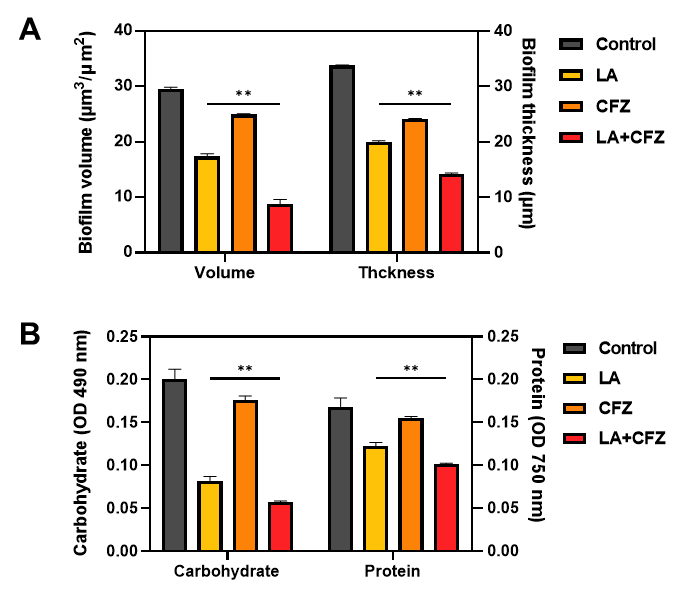


**Fig. S8.** *S. aureus* biofilm formation by the optimal combination of LA (25 μM) and CFZ (0.4 μM) under continuous conditions. (A) Biofilm volume and thickness based on CLSM images. (B) The amounts of carbohydrates and proteins in the biofilm. Biofilm was formed on silicon pads using a drip-flow reactor. (**) P<0.005 compared with the control.
